# A novel workflow for the qualitative analysis of DNA methylation data

**DOI:** 10.1016/j.csbj.2022.10.027

**Published:** 2022-10-23

**Authors:** Antonella Sarnataro, Giulia De Riso, Sergio Cocozza, Antonio Pezone, Barbara Majello, Stefano Amente, Giovanni Scala

**Affiliations:** a Department of Molecular Medicine and Medical Biotechnologies, University of Naples “Federico II”, Naples, Italy; b Department of Biology, University of Naples “Federico II”, Naples, Italy

**Keywords:** DNA methylation, Epigenetics, Epialleles, CG methylation, Non-CG methylation, Bisulfite sequencing

## Abstract

DNA methylation is an epigenetic modification that plays a pivotal role in major biological mechanisms, such as gene regulation, genomic imprinting, and genome stability. Different combinations of methylated cytosines for a given DNA locus generate different epialleles and alterations of these latter have been associated with several pathological conditions. Existing computational methods and statistical tests relevant to DNA methylation analysis are mostly based on the comparison of average CpG sites methylation levels and they often neglect non-CG methylation. Here, we present EpiStatProfiler, an R package that allows the analysis of CpG and non-CpG based epialleles starting from bisulfite sequencing data through a collection of dedicated extraction functions and statistical tests. EpiStatProfiler is provided with a set of useful auxiliary features, such as customizable genomic ranges, strand-specific epialleles analysis, locus annotation and gene set enrichment analysis. We showcase the package functionalities on two public datasets by identifying putative relevant loci in mice harboring the Huntington’s disease-causing Htt gene mutation and in Ctcf +/− mice compared to their wild-type counterparts. To our knowledge, EpiStatProfiler is the first package providing functionalities dedicated to the analysis of epialleles composition derived from any kind of bisulfite sequencing experiment.

## Highlights

• A novel R package (EpiStatProfiler) for the qualitative analysis of DNA methylation data.
• A novel workflow for the analysis of CG and non-CG epialleles starting from any type of bisulfite sequencing data.
• EpiStatProfiler can perform strand-specific characterization of epialleles composition.
• Important loci can be annotated along with their biological role and potential functions.
• EpiStatProfiler has the ability to identify loci whose epiallelic profile is associated with disease pathogenesis.

## Graphical abstract

**Figure:**
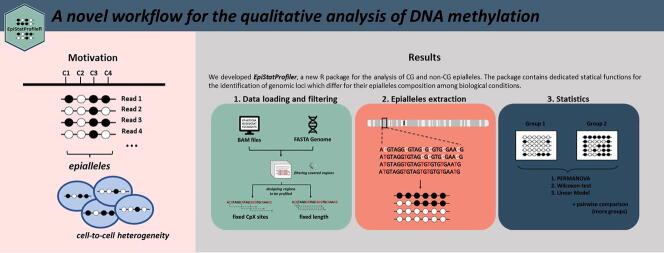

## 1 Introduction

DNA methylation is an epigenetic modification involved in major biological mechanisms, such as gene regulation, genomic imprinting, and genome stability [1], [2]. In mammals, it is mostly generated by the addition of a methyl group on the fifth carbon of the cytosine, mainly occurring in the context of CpG dinucleotides [3].

However, several recent studies have demonstrated that non-CG methylation may also exert a functional role in certain biological processes, especially in some tissues such as the brain [4], [5]. Specific DNA methylation patterns are established during cell development and differentiation, and they are stably inherited across cell cycles [6]. On the other hand, stochastic and deterministic events drive somatic DNA methylation, making it difficult to decode [7], [8]. Disruptions of DNA methylation patterns have been associated with several disease conditions, such as developmental impairment, cancer and trinucleotide repeat disorders [9], [10], [11], [12].

The gold standard to assess DNA methylation remains the bisulfite sequencing, which allows the identification of methylated cytosines at the single nucleotide level. Classical quantitative analyses then rely on the calculation of the proportion of methylated cytosines at a single position or fragment level from bulk samples. However, bisulfite sequencing techniques are also revealing greater complexity and qualitative approaches to the study of DNA methylation are emerging as complementary analysis to the classical quantitative one [13], [14], [15].

Among these latter, the epiallele-based analysis (EBA) relies on the characterization of the specific methylation patterns present on each sequenced molecule [16]. In particular, given a genomic locus containing *n* Cs in the CpX context, all the possible combinations of the methylation states of these Cs are defined as *epialleles*. This type of analysis can provide additional insights about the epigenetic cellular heterogeneity characterizing a sample [7], [17]. More specifically, the epiallele composition observed at one locus can be associated with different biological conditions. For example, it may discern the presence of different cell subpopulations (or cellular states) within the bulk sample, or it may be the expression of stochastic DNA methylation dynamics. When characterized by the presence of epiallele species with opposite methylation states, it may also depict allele-specific DNA methylation [18]. In cancer, different epiallele compositions observed at distinct stages may indicate the selection of specific tumor subclones or - conversely - the stochastic gain of aberrant DNA methylation, depending on the observed shift [7], [17], [19], [20].

**Fig. 1:**
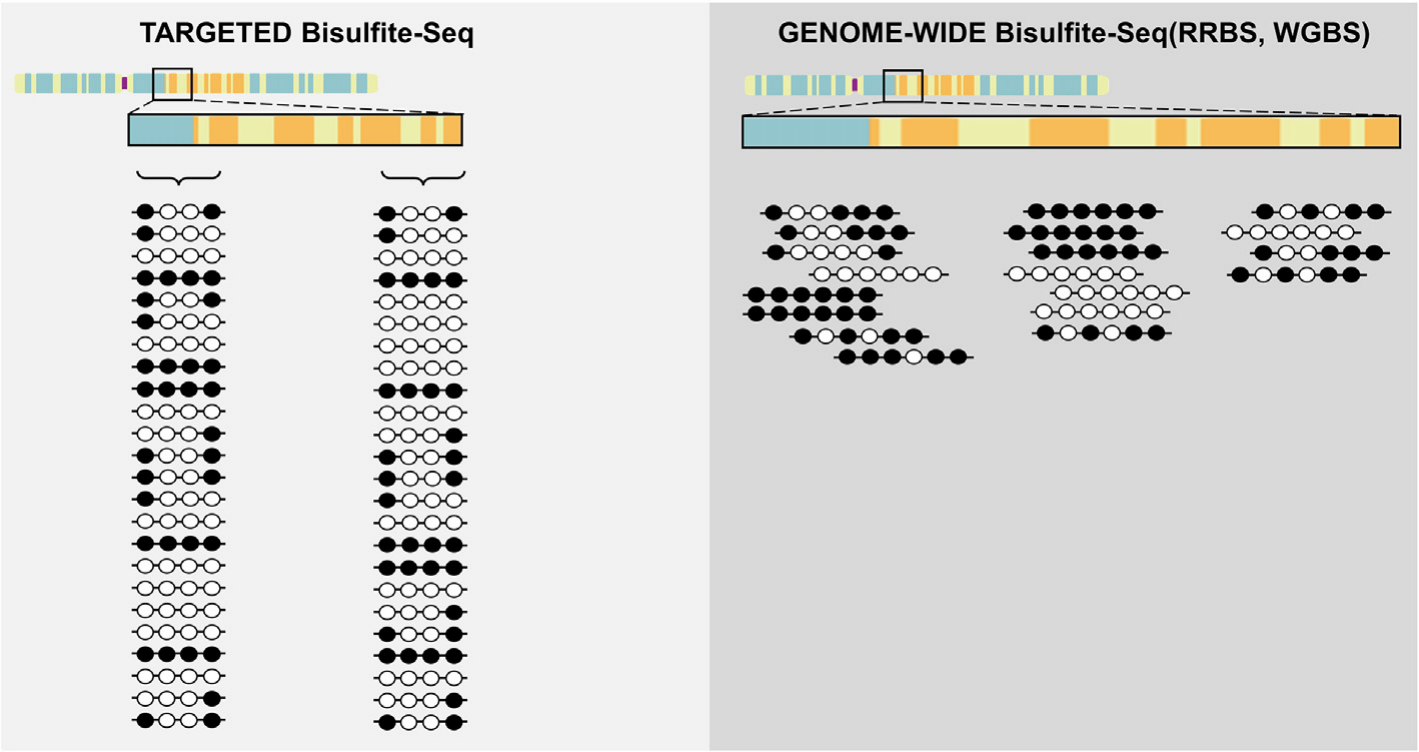
*Two approaches to study DNA methylation through the epialleles analysis.* The figure shows the two main experimental approaches currently used to retrieve the epialleles information from Bisulfite Sequencing data. The image is intended to highlight which are the advantages and the pitfalls characterizing each of these methods.

The epialleles information for a given genomic locus can be obtained from distinct types of bisulfite sequencing experiments. In particular, this kind of analysis can be either based on targeted high-coverage approaches [21] or on genome-wide low-coverage experiments [22] (see Fig. 1).

In this context, various bioinformatic tools have been developed for extracting and analyzing epialleles from targeted [8], [23] and genome-wide bisulfite sequencing data [19], [24].

The tools based on targeted DNA methylation allow the analysis of methylation profiles from deep targeted bisulfite sequencing. This approach relies on the high-resolution levels obtained from targeted bisulfite experiments and provides an in-depth characterization of epialleles species for one or more targeted genomic regions and the possibility to represent and analyze the epiallele composition using population genetics-based approaches [8], [23]. The main limitation of these tools is that they can only perform the analysis on a particular type of input data. Moreover, raw data from deep targeted sequencing are not easily found in public databases, thus making it difficult to re-use public methylation datasets.

On the other hand, the tools that overcome these limitations by extracting information from genome-wide experiments - such as Methclone [19] – lack statistical functions for comparing epiallele composition between different groups of samples. This latter aspect is crucial when the focus of the analysis goes beyond the simple characterization of epiallele families for one or more regions in one or more samples. Indeed, this kind of data can be used to compare epiallele distributions between one or more sample groups associated with different biological conditions and to locate genomic regions whose epiallelic compositions vary between conditions, thus leading to the discovery of novel biomarkers that can be missed using classical quantitative approaches.

In this manuscript we present EpiStatProfiler, a new R package providing a set of functions that allow a customized genome-wide analysis and statistical comparison of epialleles composition within and among different groups. In particular, EpiStatProfiler has been developed with the ability to extract epialleles information from any type of bisulfite sequencing data, starting from genomic intervals that can be easily designed by the user in a modular fashion. EpiStatProfiler also enables the analysis of non-CG methylation, letting the user define the CX mode used to estimate the methylation patterns in a given locus. Moreover, EpiStatProfiler is provided with the flexible functionality to perform a strand-specific characterization of epialleles composition. This kind of analysis is necessary when characterizing non-CG methylation, since these other modifications - *per se* - are not symmetric, but it may also be helpful to dissect strand-specific methylation in the CpG context, already described in literature [25].

Furthermore, the package contains a collection of statistical functions that allow the identification of loci which differ among groups for their epialleles composition or for an extensible set of epiallele-derived summary statistics (e.g. Shannon Entropy).

Finally, we demonstrate the package functionalities by identifying putative relevant loci in two different experimental designs.

## 2 Methods

### 2.1 RRBS raw data processing

Raw RRBS data used to demonstrate the package were downloaded from SRA using the GEO accession GSE147156 (considering only the samples collected from the striatal tissue) and GSE48975. FastQ files were processed using an in-house pipeline. Data were quality-checked by using FastQC v0.11.9 (https://www.bioinformatics.babraham.ac.uk/projects/fastqc/). Low-quality bases and adapters were removed by using Trim Galore v0.6.6 with --rrbs option (https://www.bioinformatics.babraham.ac.uk/projects/trim_galore/). Selected reads were then aligned to the mm10 reference genomes by using Bismark v0.23.0 using default parameters. BAM files were finally sorted and indexed using the SAMtoolsKit (https://www.htslib.org/).

### 2.2 Epialleles extraction algorithm implementation

**Fig. 2:**
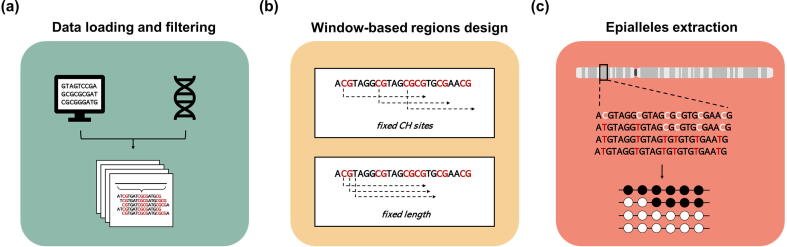
*EpiStatProfiler workflow: epialleles extraction.* The figure shows the first three modules of the EpiStatProfiler workflow. In the left part (a) the data loading and filtering are described. BAM files are loaded along with the reference genome file and then are filtered to select only covered loci. In the second step (b) the user defines the regions where to perform epialleles profiling. This can be achieved using one of the two different approaches shown in the Figure. The last module (c) consists in the extraction of epialleles composition from each of the regions obtained from the previous step. Two different outputs are created in this step: the compressed epialleles matrix, containing the epialleles counts for each region, and a summary data frame containing a set of user-defined epialleles-based statistics.

To facilitate the analysis, one function has been designed to select the genomic regions covered by a sufficient number of reads in the BAM files. First, the coverage is calculated genome-wide at single-base resolution, and then the contiguous regions satisfying a user-defined coverage threshold are merged to generate a collection of genomic ranges with different lengths. The subsequent preliminary step of the workflow consists in the partitioning of covered areas into a group of regions, each one constituted by a contiguous set of covered sites meeting user-defined criteria as described below. Two distinct approaches have been implemented to achieve this step. In the first case, genomic intervals are obtained using sliding windows of flexible length containing a user-defined number of CpX sites, stepping up by 1 CpX site at time. Alternatively, target regions are generated using a sliding window with a user-defined fixed length and step sizes, containing a user-defined minimum and maximum number of CpX sites. Since the identified methylation patterns are derived from single fragments, the maximum length of the regions depends on the read length of the sequencing experiment. For each region to be profiled, epialleles composition is extracted as described below (see also Fig. 2). First, all the sequenced reads mapping to the corresponding locus are selected. These reads are processed separately, according to the strand on which they are aligned, and their sequence is then compared with the reference genome at the position of the CpX sites. Given the number of Cs in the CpX context at one locus (n Cs) and the number of reads spanning the entire region taken into account (n Reads), a table - composed of n Cs columns and n Reads rows - is compiled row-wise as follows. For each read and each CpX position, if a T (or an A on the reverse strand) is found in correspondence of the reference C position, then the corresponding position in the table is marked as 0 (unmethylated), otherwise as 1 (methylated). Depending on the user choice, epiallele composition from different strands can be analyzed separately, thus allowing a stranded epiallele estimation, or it can be evaluated independently of the originating strand.

### 2.3 Bisulfite conversion quality check

To ensure that the status of cytosines methylation is properly assessed in the analyzed regions, the bisulfite conversion efficiency can also be monitored. In all the experiments where non-CG methylation can be considered as a minor phenomenon, it can be assumed that all the cytosines that are not in the CG context are converted to thymines upon bisulfite treatment. Thus, bisulfite efficiency is calculated for each sequenced molecule as the percentage of CHH cytosines (except those Cs in the CG context) that have been converted among all the CHH cytosines that are located in the covered genomic interval. The reads with low conversion efficiency can be optionally removed from the epiallele matrix. Then, the coverage for each interval is recalculated and only those regions that meet the user requirements are retained for the analysis. The same rationale is used when the pattern examined through the epialleles analysis is different from “CG”, but in this case the cytosines in the CG context are not considered to calculate the ratio of cytosines subjected to conversion.

### 2.4 Ambiguous reads and polymorphisms

The presence of ambiguous reads is also tracked during the extraction step. When filling the binary epialleles matrix, if nor a T (or A) or a C is found in the correspondence of a CpX site, the corresponding cell is filled with the value of 2. These reads are then excluded while extracting the epialleles information.

### 2.5 Output generation

Two different outputs are generated starting from the binary epiallele matrix related to one locus. The first one is a compressed epiallele table. To build this output, each row of the binary epiallele matrix is converted to a string that represents one epiallele species. The compressed table is then created by reporting the count for each epiallele species found at the analyzed locus. The second output is obtained by applying a customizable set of functions that take the binary matrix as input and compute, each one, a given summary statistic. A data frame that contains the computed summary statistics is then generated. The number and the type of functions to compute summary statistics over the epiallele binary matrix can be easily changed and extended with user defined functions.

### 2.6 Available summary statistics

The following predefined set of summary statistics have been implemented in EpiStatProfiler.

#### 2.6.1 Number of CpX sites

The number of CpX sites (*n*) defines the set of CpX positions used to retrieve the epialleles information in one analyzed region. This is useful when using sliding windows of the same length containing a variable number of CpX sites. It can be used to normalize other statistics.

#### 2.6.2 Number of reads spanning the entire locus

The number of reads (*c*) indicates the number of sequenced reads that entirely cover the analyzed locus.

#### 2.6.3 Mean distance between CpGs

The average distance between the cytosines in the CpX context (expressed in bp) defines at which extent cytosines are aggregated in the analyzed region. It is calculated using the following formula:

$$d=\left(\overset{n}{\underset{i=1}{\sum }}(p_{i}-p_{i+1})\right)/n$$

n = number of CpX − 1.

p = position (bp) of the CpX site in the analyzed region.

#### 2.6.4 Observed epialleles species

The number of observed epialleles is also reported. This is calculated by counting the number of the unique epialleles observed in the analyzed region.

#### 2.6.5 Singleton

This function returns the number of epialleles species found in a single copy among the sequenced reads associated with the region. This latter is informative about the number of rare epialleles that are found in the analyzed region.

#### 2.6.6 Epiallele with the highest frequency

The epiallele with the highest frequency is obtained by reporting the string associated with the highest count in the epialleles matrix.

#### 2.6.7 Shannon entropy

The Shannon entropy is used as a measure of probabilistic uncertainty. Having the epialleles composition, the Shannon entropy estimates how heterogeneous is the population of the epialleles species at one locus, with values ranging from 0 (when all the reads carry the same epiallele) to *n_CpX* (when all possible epiallele states are found with equal frequency).

Given *n_CpX* sites in the region, the Shannon entropy is calculated as:

$$\text{H = -}\sum_{\text{k=1}}^{\text{e}}{\text{p}}_{\text{k}}\text{*}\;{\text{log}}_{\text{2}}{\text{p}}_{\text{k}}\;$$

where *e* is the maximum number of distinct epiallele species that can be observed in one locus (${\text{2}}^{\text{n CpX sites}}$) and *p_k_* represents the frequency of the k-th epiallele.

#### 2.6.8 Average DNA methylation

Given the population of epialleles in one genomic region, the average DNA methylation in the region is also calculated. This is done dividing the number of methylated cytosines (m) in the binary matrix by the total number of cytosines in all the sequenced reads.

a = m/n∗c

*n* = number of CpX sites in the locus.

*c* = sequenced reads covering the locus.

### 2.7 Statistical testing

The comparison of epialleles compositions among groups at multiple genomic regions is performed using PERMANOVA, a non-parametric multivariate statistical test. Having the epiallele composition matrix at one locus for each sample, this analysis is based on the prior calculation of distances between each pair of samples in the dataset. The test calculates whether the distances among samples belonging to different groups are higher than the distances found among samples within the same group. The significance of the result is then computed by calculating the chances that the same difference observed within sample data can also be obtained by randomly allocating the samples to the different groups through a series of *n* permutations. Finally, a p-value is assigned to each analyzed genomic region. Adjusted p-values are also calculated using the *fdr* method. The test has been implemented using the *adonis2* function from the *vegan* R package [26], which provides tools for descriptive community ecology. A post-hoc analysis is performed when comparing the epialleles composition matrices among more than two groups, and this is achieved by using the pairwise.adonis R package. Dimensionality reduction is also performed to visualize data using the Principal Component Analysis (PCA) and the Canonical Correspondence Analysis (CCA).

To compare the distributions of user defined summary statistics, a non-parametric test is adopted to assess whether the difference among the groups is statistically significant. In this case a non-parametric test was used since the distribution of the test variable cannot be predicted *a priori*. The Wilcoxon test is used to compare genomic regions when only two groups are available, otherwise Kruskal-Wallis test is adopted. Finally, to compare mean values of summary statistics considering a covariate (e.g., time), an analysis of covariance (ANCOVA) is implemented.

### 2.8 Association with genes

Differentially heterogeneous regions are associated with closest genes in EpiStatProfiler using the regsToPathway function. This latter is based on the *annotatePeak* function from the *ChIPseeker* R package [27]. Each region is annotated considering the regions ± 1000 kb flanking the TSS of genes as promoters. The annotation of each region - based on the relative localization to its closest gene - can be: “Distal intergenic”, “Promoter”, “Exonic”, “Intronic”, “3’ UTR”, and “5’ UTR”.

### 2.9 DMP (Differentially methylated Positions) detection

The quantitative analysis of DNA methylation was performed by using the R package *methylKit* [28]. Methylation calls were performed starting from the aligned bam files using the *processBismarkAln* function, by considering only CpG sites covered by at least 10 reads and present in at least 3 samples per group.

Differentially methylated positions were detected by calling the *calculateDiffMeth* function and by selecting the sites having a q.value ≤ 0.05 and an absolute average methylation difference ≥25 %.

### 2.10 Enrichment analysis for genomic regions

To test the enrichment of CTCF binding sites within the set of significant genomic regions obtained from the analysis of dataset 2 (GSE48975), we first retrieved CTCF ChIP-seq peaks of mouse lung tissues from two ENCODE assays (ENCFF491RJK, ENCFF605YVN), then we generated a bed file containing only the overlap between the peaks from the two experiments and finally passed it along with the bed files containing significant regions to the *bedtools fisher* tool.

## 3 Results

Here we introduce EpiStatProfiler, a novel workflow for the qualitative analysis of DNA methylation from bisulfite sequencing data. It contains a set of functions dedicated to the extraction and statistical analysis of epialleles derived from bisulfite sequencing experiments. EpiStatProfiler is distributed as an open-source R-package and is publicly available on GitHub (https://github.com/BioinfoUninaScala/epistats).

### 3.1 Input data loading

The first module is dedicated to input data filtering and to the design of the regions to be profiled. Input data required by EpiStatProfiler is represented by aligned BAM files derived from bisulfite sequencing experiments, along with a reference genome file in FASTA format. To restrict the assessment of the epiallele composition to regions with sufficient read coverage, the genomic regions from the bam files are filtered based on a user-defined coverage threshold, as described in the Methods section. Alignments can be optionally restricted to a subset of chromosomes of interest from the experiment by using dedicated functions. At the end of the filtering steps, the regions satisfying user parameters are converted to a GenomicRanges object.

### 3.2 Using sliding windows to design target regions

The target regions can be designed based on several user-defined criteria. First, it must be decided which type of sliding window system should be used to construct the regions. The package has two distinct functions that correspond to two different approaches described in the Methods section. Regardless of the chosen system, the user must set the pattern to be searched for within the genome to build up the analysis window set. If the user is interested in CG methylation, this parameter will be equal to 'CG', otherwise one of 'CA', 'CC' or 'CT'. The functions that build these regions have also been implemented to return the genomic coordinates of the analyzed cytosines.

### 3.3 Extracting epialleles composition

The epiallele composition is extracted from each region obtained in the previous step. Before executing the extraction, the user can fine tune different parameters guiding the extraction step. More specifically, the user can decide whether to apply further filters on the input data, such as the removal from the epiallele composition of those reads that do not satisfy a certain threshold of bisulfite conversion. By the end of the extraction step, two different outputs are returned for each analyzed region. The first one is a compressed matrix that represents the epiallele composition by storing the number of occurrences of each epiallele in that region. The second is a data frame containing as many columns as the number of different summary statistics calculated on the raw binary epiallele matrix as input. The user can pass to the function a list of one or more statistics chosen from those provided within the package and can further provide user-defined functions implementing other statistics of interest. Finally, a third output containing the regions excluded from the analysis is generated. Each of these described outputs is locally saved as a text file.

### 3.4 Dedicated statistical module

Once the epiallele composition of all the passing filter regions from each sample of the dataset is available, the analysis can continue with the computation of one or more statistical functions provided with the package aimed at identifying regions whose epiallele composition differs among sample groups. These functions can be divided into two groups: a first set of functions working directly on the epiallele matrices and a second setworking with the computed summary statistics.

#### 3.4.1 Functions working on epialleles matrices

**Fig. 3:**
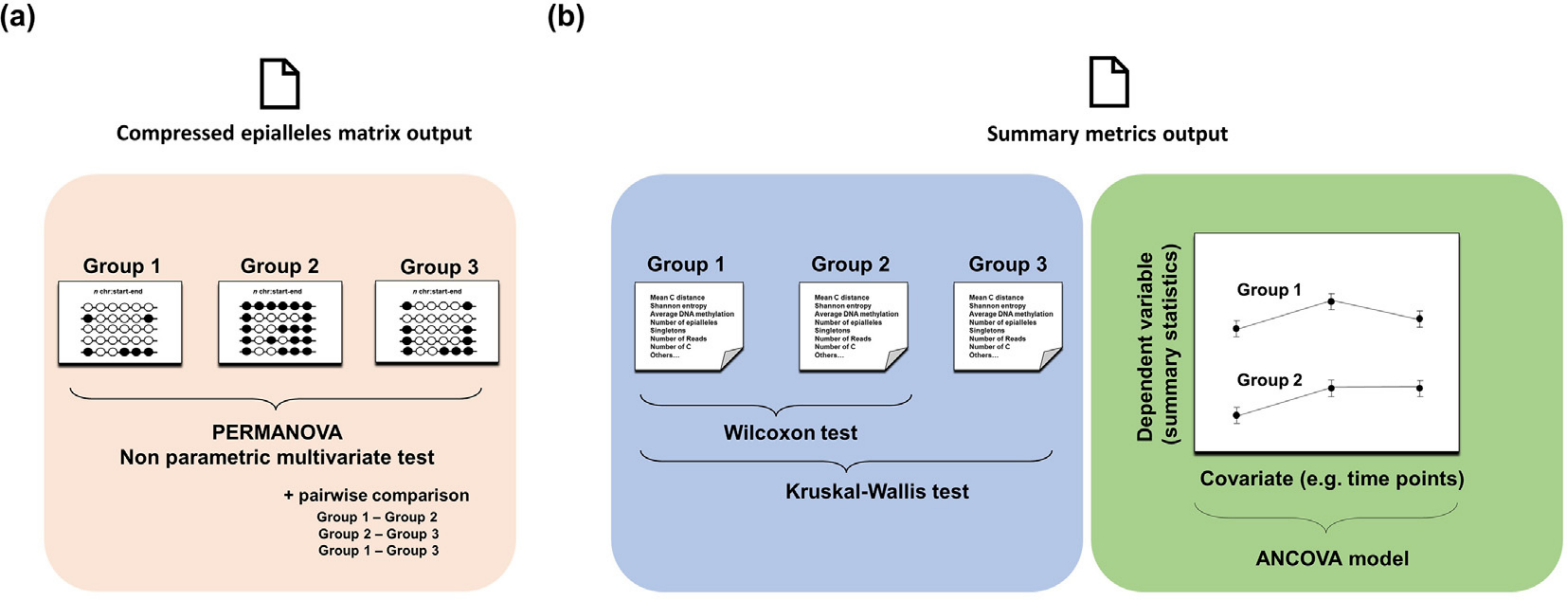
*Statistical module.* The figure shows the main statistical tests implemented in EpiStatProfiler to compare epiallele composition among/between groups. The left panel (a) shows the test statistics used to compare the raw epialleles counts among different groups. In this case a non-parametric multivariate test (PERMANOVA) is adopted. In the right panel (b) are described the tests used to compare the epiallele-based summary statistics among/between groups. The first type is a non-parametric test. The choice of the test (Wilcoxon vs. Kruskal-Wallis) depends on the number of groups provided by the user. The second one is a linear model which performs an analysis of covariance.

The functions working on the count matrices (Fig. 3a) consider the epialleles observed in one region as a population of individuals (represented by the set of reads covering that region) belonging to different species (represented by the different epiallelic conformations). PERMANOVA, described in the Methods section, is implemented in EpiStatProfiler in the *epiStat* function. The input data needed to run these functions are the list of the epialleles matrices from all the samples and a table containing sample metadata. The user must then indicate which columns are the ones containing the group and the sample information. Additional parameters can be set to perform the analysis, such as the minimum number of samples in each group required to carry out the analysis. The function returns as output a data frame containing all the regions analyzed as rows, along with the results of the statistical test.

Additional functions are provided to perform tests on single regions data. The *pairtest* function can be used to perform the pairwise comparison (see Methods) when more than two groups are present, in order to detect which group is mostly contributing to the dissimilarity observed in the tested locus. Finally, a function is provided to identify the epiallele species driving the dissimilarities between the groups in each genomic region. Both these functions require as one parameter the ID of the region to be analyzed.

#### 3.4.2 Functions working on summary statistics

The package contains two functions (Fig. 3b) that compare the summary statistics values among groups: *diffStat* and *diffModel*. These latter accept as input the table containing the summary statistics for each analyzed interval. The first one is aimed at identifying the regions which differ among the provided groups for a given statistic. The user can indicate which statistic has to be selected to perform the test. The output is a table containing the IDs of each region and the result of the test, this latter reported through the (adjusted) p-value. Additional information is also added to the output, such as the name of the test used to perform the analysis (see Methods section) and the median values of the input statistic for each group.

On the other hand, the *diffModel* function performs the analysis of covariance (ANCOVA) described in the Methods section. This can be useful to evaluate whether the groups differ for one statistic across different levels of another variable (e.g., different time points). The dependent variable and the covariate can be provided by the user through the function parameters. The output consists of a table containing the IDs of each region, along with the result of the statistical test, the p-values referring to the effects of single variables taken alone and those related to their interaction terms. The coefficients of the fitted model are also reported in the output, as well as the slopes of the regression lines for each group.

The results obtained by applying these two sets of functions have different and complementary biological relevance. The functions handling epiallele composition allow the comparison of general differences in the epiallele population. Summary statistics, on the other hand, can be used to compare specific aspects of epialleles populations such as epialleles clonality, richness or the presence of rare epialleles.

### 3.5 Results visualization

Once one or more regions of interest are selected, the user can run functions provided by EpiStatProfiler to plot the epiallelic information related to these regions. Two different functions are implemented in the package to visualize data by obtaining ordination plots for each locus. They perform the two ordination analyses described in the Methods, the PCA and CCA respectively. Both functions take as input the compressed epiallele matrices along with the samples metadata and the ID of the region of interest, returning the ordination plot.

### 3.6 Functional analysis

Finally, to investigate the biological functions eventually linked to the identified differences in epiallele composition, EpiStatProfiler is provided with the *regsToPathway* function to associate significant regions with genes. The user just needs to specify the reference genome to be used for the association analysis. The output of this function is a table containing several information related to each region-gene association, such as the distance to TSS, the type of association (Exonic, Intronic, Intergenic, etc…) and the gene symbol. This information can then be used to perform further downstream analyses, such as gene enrichment analysis (see Case of Study below).

### 3.7 Case of study

To showcase the EpiStatProfiler workflow, we report the analysis of two datasets consisting of RRBS and enhanced RRBS data from two distinct experimental designs.

The first dataset comprises heterozygous *Htt* knock-in (**Q175**) and wild-type (**Q20**) mice [29], derived from an experiment designed to identify putative methylation signatures in mouse models carrying the causative mutation of the Huntington disease.

The input dataset consists of 8 different samples from each group, retrieved from the striatal tissue of 3 weeks old mice. We processed the raw fastq files (see Methods) and then, starting from aligned bam files, we implemented the EpiStatProfiler workflow as described below.

**Fig. 4:**
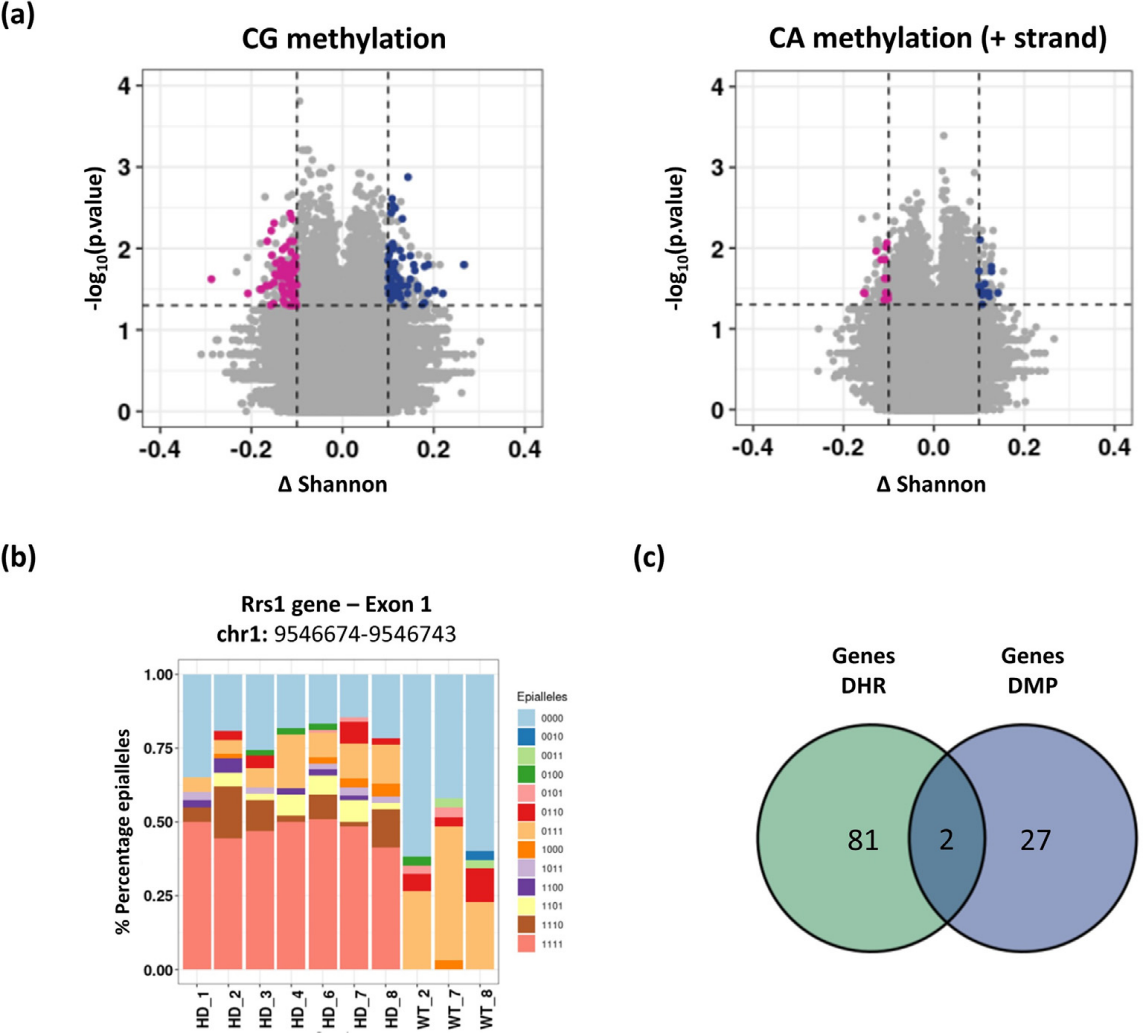
*Description of the significant differentially epigenetic heterogeneous regions found in CG and CA contexts*. (a) Relevant loci of HD mice are shown using volcano plot. Loss and gain in clonality composition are marked in violet-red and blue respectively. The relevant loci were obtained by filtering the regions having a p.value ≤ 0.05 and being characterized by an absolute median Shannon entropy difference ≥0.10 between the two groups and further selected for being characterized by a significantly different epialleles composition among the two groups (PERMANOVA p.value ≤ 0.05). (b) A significant region obtained from the filtering processes used as proof of concept. Regions coordinates and functional annotation are reported on the top of the barplots. (c) Overlap of the genes associated with significant regions obtained performing the qualitative (DHR = differentially heterogeneous regions) and the quantitative (DMP = differentially methylated positions) analyses, respectively, within the classical CG context. (For interpretation of the references to colour in this figure legend, the reader is referred to the web version of this article.)

We first selected all the genomic regions covered (end to end) by at least 30 reads in each of the input Bam files and we then designed the target regions using EpiStatProfiler in the “sliding window” mode with a fixed window length of 70 bp (based on the average read length of this specific sequencing experiment). We executed the workflow to extract epialleles composition in both the CG and CA contexts. To identify the genomic regions whose epiallele composition differs in terms of clonality between the HD and the wild-type samples we used the *diffStat* function and compared the Shannon entropy values between the two conditions by using a Wilcoxon non-parametric test. The statistically significant loci were obtained by filtering the regions having: i) a p.value ≤ 0.05 and ii) an absolute median Shannon entropy difference ≥0.10 between the two groups (Fig. 4a). Overall, we found 370 significant regions that underwent a significant Shannon entropy change in the HD group when considering the CG context and 183 significant regions when looking at the CA methylation (all belonging to the *plus* strand).

We further filtered the significant regions, by retaining only those ones which were also found to be significantly different by running the *epiStat* function, which directly compares the epialleles composition among groups. In this way, we identified 135 regions (colored dots in Fig. 4a, CG methylation) characterized by either different epiallele composition and epigenetic clonality (measured through their Shannon entropy) among groups in the CG context, along with 30 regions in the CA context (colored dots in Fig. 4a, CA methylation). As proof of concept, we reported as example one significant region which shows an increased Shannon entropy in the HD group and a significantly different epiallele composition compared to the wild-type group (barplots in Fig. 4b). This latter region is located in the first exon of the Rrs1 gene, whose altered expression is commonly described in knock-in mice models of HD disease [30].

Next, we associated these significant regions with genes (coding and non-coding) and retained only those (n = 102 for CG context and n = 11 fort CA context) annotated in functional domains (Promoter = 45, Intron = 25, Exon = 26, UTRs = 6 in CG context and Promoter = 1, Intron = 8, Exon = 2 in CA context). We then used the genes found to be associated with our significant regions in both CG and CA contexts (CG genes = 83, CA genes = 11) to perform a differential gene set enrichment analysis using *g:Profiler* [31] to identify specific biological processes. We found significantly enriched GO biological terms implicated in the disease pathogenesis looking at both CG and CA methylation signatures (see Table 1) [32], [33].

**Table 1:** Results of the gene set enrichment analysis (Dataset 1).

	Source	Term name	Term ID	Adjusted p.value
CG methylation	GO:BP	embryonic morphogenesis	GO:0048598	2.100 × 10^−2^
CG methylation	GO:BP	cell differentiation	GO:0030154	3.949 × 10^−2^
CG methylation	GO:BP	cellular developmental process	GO:0048869	4.500 × 10^−2^
CG methylation	GO:BP	regulation of cellular metabolic process	GO:0031323	4.661 × 10^−2^
CG methylation	GO:BP	cerebellar molecular layer development	GO:0021679	4.980 × 10^−2^
CA methylation	KEGG	Glutathione metabolism	KEGG:00480	3.058 × 10^−2^
CA methylation	WP	Oxidative stress and redox pathway	WP:WP4466	1.880 × 10^−2^

**Fig. 5:**
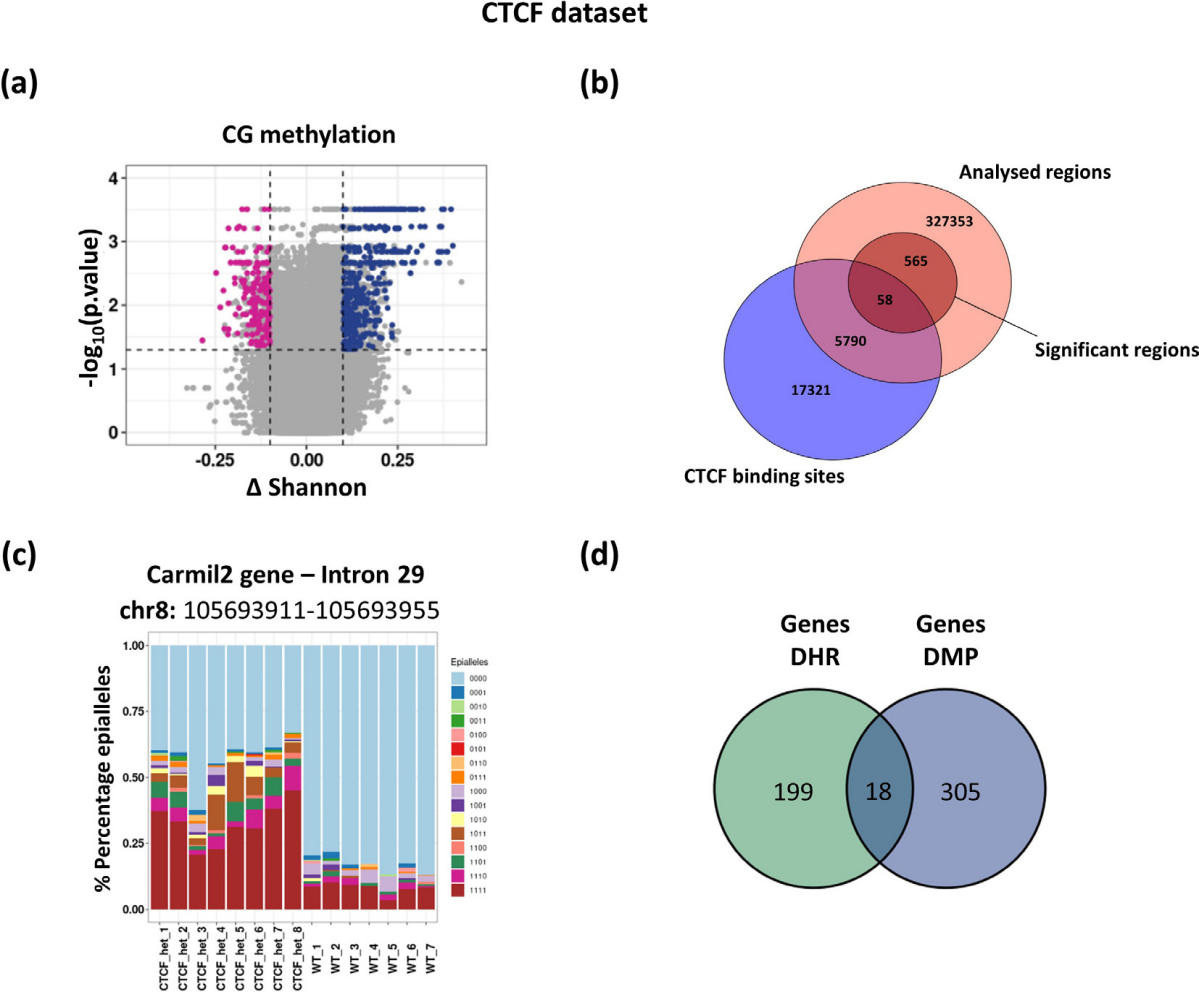
*Description of the significant differentially epigenetic heterogeneous regions found in CG contexts in dataset 2 (wild-type and Ctcf +/− samples)*. (a) Differentially heterogeneous epigenetic regions between wild-type and Ctcf +/− mice are highlighted using a volcano plot. Loss and gain of epigenetic heterogeneity are marked in violet-red and blue respectively. The relevant loci were obtained by filtering the regions having a p.value ≤ 0.05 and being characterized by an absolute median Shannon entropy difference ≥0.10 between the two groups and further selected for being characterized by a significantly different epialleles composition among the two groups (PERMANOVA p.value ≤ 0.05). (b) Scaled Venn diagram showing the enrichment of CTCF binding sites in regions showing statistically significant epiallele heterogeneity between the wild-type and Ctcf +/− samples. (c) Barplots showing the proportion of observed epialleles (different colors) in all the analyzed samples in a selected significant region (coordinates and annotation shown on the top of the barplots). (d) Venn diagram showing the overlap between the genes associated with significant regions from the qualitative (DHR = differentially heterogeneous regions, green) and the quantitative (DMP = differentially methylated positions, blue) analyses within the CpG context. (For interpretation of the references to colour in this figure legend, the reader is referred to the web version of this article.)

We then followed the same workflow to analyze a dataset consisting of whole lung tissue samples from *Ctcf* hemizygous knockout (*Ctcf* +/−, n = 8) and wild-type (n = 7) mice. To identify genomic regions undergoing epialleles heterogeneity alterations between the *Ctcf* +/− and the wild-type mice we used both the *diffStat* and the *epiStat* functions, as described above. Here, we could identify 623 significant regions which differed for their Shannon entropy and their epialleles composition between the two conditions in the CpG context (Fig. 5a), with most of them (n = 435/623, in blue in Fig. 5a) showing an increase of Shannon entropy levels in *Ctcf* +/− depleted samples (as in Fig. 5c).

We then associated the above reported significant regions with genes and retained only those (n = 549 for CG context) annotated in functional domains (Promoter = 97, Intron = 88, Exon = 227, UTRs = 137). We then used the genes found to be associated with our significant regions (CG genes = 217) to perform a differential gene set enrichment analysis using *g:Profiler* [31] (Supplementary File 1) [31]. Of note, we could identify an enrichment of genes described as CTCF targets. To better explore this observation, we assessed whether the obtained significant genomic regions were enriched for CTCF binding sites. In particular, we tested their overlap with CTCF-binding regions derived from mouse whole lung tissues from ENCODE and found a statistically significant enrichment (Fisher’s exact test p.value < 2.5078e-76, Fig. 5b).

Finally, to compare our results with those obtained from a classical quantitative analysis, we performed a differential methylation analysis in both datasets (see Methods). In particular, we analyzed cytosines in the CpG context covered by at least 10 reads whose methylation status could be assessed in at least 3 samples per group. We then selected differentially methylated positions by filtering the sites showing a qvalue ≤ 0.05 and a methylation difference percentage > 25 % among the two groups (WT and HD, WT and Ctcf +/−). Referring to the canonical CG context, we evaluated the overlap of the genes associated with the significant regions identified through the two different analyses (Genes DHR, Genes DMP). We found that the two approaches detected significant loci that had some overlaps, showing that the qualitative analysis can capture additional methylation changes events that could be missed by standard DNA methylation analyses (Fig. 4c, Fig. 5d) [8].

## 4 Discussion

In this manuscript we presented EpiStatProfiler, an R package for the qualitative analysis of DNA methylation sequencing experiments. A plethora of methods aimed at identifying genomic regions showing differential DNA methylation patterns among different biological conditions have been proposed in literature, with most of them relying on a quantitative view of the observed methylation levels for a given locus and their tests based on the comparison of bulk-average DNA methylation levels.

A more recent approach to the study of this modification is based on a qualitative evaluation of DNA methylation patterns, relying on the possibility to characterize specific DNA methylation patterns at the single molecule resolution. Given a genomic locus containing individual CpX sites, and starting from a bisulfite sequencing experiment, it is possible to estimate the distribution of the distinct methylation patterns (*epialleles*) that can be observed among all the possible ones. The most relevant aspect of this characterization is that it can be used as a proxy to evaluate the cell-to-cell epigenetic heterogeneity within a sample; an information that is often poorly covered within standard quantitative analysis.

We developed a comprehensive tool to support the analysis and statistical comparison of the epiallele composition within and among samples in the R environment.

Compared with existing tools (see Table 2), EpiStatProfiler provides additional functionalities improving the extraction of epialleles and offer various statistical tests to identify relevant loci that could be further investigated as putative epigenetic biomarkers. Indeed, most of the existing tools (see Table 2) are not exhaustive in the set of analysis they carry out and they lack dedicated statistical tests for the comparisons of epialleles composition between different groups for a given experimental design.

**Table 2:** Comparison of existing tools which perform epialleles extraction from bisulfite sequencing data.

	Programming language	Input data	Type of experiment	Stranded analysis	Non-CpG methylation	Statistics	Bisulfite conversion QC
ampliMeth Profiler	*Python*	FASTA files	Targeted	No	No	Yes	Yes
mHapTools	*C, Command line*	BAM files	Genome-wide	No	No	No	No
methclone	*C ++*	BAM files	Targeted, Genome-wide	No	No	No	Yes
EpiStat Profiler	*R*	BAM files	Targeted, Genome-Wide	Yes	Yes	Yes	Yes

EpiStatProfiler has been designed to extract epialleles information from any kind of bisulfite sequencing data, whereas tools such as AmpliMethProfiler and Methclone have been mainly developed for specific types of experiments (targeted vs genome-wide, respectively).

Compared to the other tools and in relation to the possibility to analyze different type of bisulfite sequencing experiments, EpiStatProfiler provides dedicated functions to select, filter and analyze subset of genomic regions from the whole assay. This is especially useful when dealing with wide low-coverage assays (like WGBS and RRBS) to retrieve only regions of interest characterized by a sufficient quality and depth.

In contrast, AmpliMethProfiler has been specifically developed to analyze amplicon-based bisulfite sequencing data, retrieving the epialleles composition from one or few target regions, while Methclone has the ability to filter genomic regions covered by a minimum number of reads but it limits the analyses to a maximum amount of 10 CpGs per region. Moreover, Methclone does not allow the specification of custom window size for the region selection (which could be useful when analyzing long-read sequencing experiments).

Furthermore, EpiStatProfiler implements some additional functionalities which are not foreseen in the other approaches indicated in Table 2. EpiStatProfiler workflow is indeed provided with the option to extract epialleles based on non-CpG sites. Other types of DNA modification, such as CA methylation, have been described to exert an important biological role in some systems and, particularly, in brain tissues. EpiStatProfiler allows the analysis of epialleles composition in these contexts, a feature that has never been described in literature to our knowledge, thus providing the opportunity to investigate strand-specific DNA methylation. Furthermore, compared with other tools, EpiStatProfiler is intended to carry out the analysis starting from any type of experimental design. Methclone can identify genomic regions harboring large changes in the clonality of their epialleles by calculating the combinatorial entropy of the epialleles distribution observed in two samples only (Diagnosis and Relapse). On the other hand, EpiStatProfiler is provided with statistical tools aimed at the identification of regions with different epialleles composition in two or more sample groups or time points.

By showcasing the EpiStatProfiler workflow in the identification of epigenetic signatures in the RRBS-based dataset, we demonstrated that this kind of approach can identify epigenetic signatures within regions associated with genes implied in disease pathogenesis in Huntington mice models. The enrichment results showed that the analysis of the CA and CG contexts is able to capture different sets of GO biological terms associated with the disease pathogenesis (see Table 1).

Indeed, among the enriched terms, we found biological pathways which are widely described as dysregulated in Huntington’s models [32], [33], [34], [35] (see Table 1). In particular, regarding the CG context, we found significantly enriched GO biological terms associated with developmental processes [32], [34], such as “embryonic morphogenesis” and “cell differentiation”, along with more specific pathways involved in the pathogenesis of Huntington’s disorder, such as “cerebellar molecular layer development” [35] (see Table 1, CG Methylation). In addition, we also found enriched terms sustained from the genes obtained from the CA methylation analysis, and these were also associated to specific pathways, in particular the “glutathione metabolism” pathway, whose dysregulation has been previously described in Huntington's disease knock-in striatal cells [33] (see Table 1, CA methylation).

Furthermore, we were able to identify genomic regions showing significant changes in their epiallele heterogeneity in lung tissue of mice characterized by the genetic disruption of one Ctcf allele. The obtained results show that significant regions are characterized by an increased epiallele entropy when Ctcf is depleted, which is compatible with other observations describing the ability of CTCF to decrease cellular expression heterogeneity by stabilizing promoter-enhancer interactions [36], [37]. Finally, the enrichment analysis of these genomic regions revealed that they are significantly enriched for CTCF-binding sites, suggesting the existence of specific dynamics of cellular heterogeneity disruption when Ctcf is depleted [36].

In conclusion, EpiStatProfiler represents a valuable tool for the characterization of the epialleles composition in various biological systems supporting researchers in exploring epigenetic information from a complementary point of view. EpiStatProfiler is available at https://github.com/BioinfoUninaScala/epistats.

## Declaration of Competing Interest

The authors declare that they have no known competing financial interests or personal relationships that could have appeared to influence the work reported in this paper.
